# Dynamic Beam Steering and Focusing Graphene Metasurface Mirror Based on Fermi Energy Control

**DOI:** 10.3390/mi14040715

**Published:** 2023-03-23

**Authors:** Sanghyeok Yu, Youngsoo Kim, Eunso Shin, Soon-Hong Kwon

**Affiliations:** Department of Physics, Chung-Ang University, Seoul 06974, Republic of Korea; shyu9922@gmail.com (S.Y.); youngsoo.kim94@gmail.com (Y.K.); ensoshin21@gmail.com (E.S.)

**Keywords:** metasurface mirror, metamaterial mirror, graphene, beam steering, focus control

## Abstract

Beam steering technology is crucial for radio frequency and infrared telecommunication signal processing. Microelectromechanical systems (MEMS) are typically used for beam steering in infrared optics-based fields but have slow operational speeds. An alternative solution is to use tunable metasurfaces. Since graphene has gate-tunable optical properties, it is widely used in electrically tunable optical devices due to ultrathin physical thickness. We propose a tunable metasurface structure using graphene in a metal gap structure that can exhibit a fast-operating speed through bias control. The proposed structure can change beam steering and can focus immediately by controlling the Fermi energy distribution on the metasurface, thus overcoming the limitations of MEMS. The operation is numerically demonstrated through finite element method simulations.

## 1. Introduction

Beam steering technology is one of the most important topics in the field of radio frequency (RF) signals [1,2] or 5G mobile communication signal processing [3,4] using gigahertz optics. The beam steering in an optical system can be easily implemented by changing the relative phase of a wave, which can be easily obtained by changing the refractive index or controlling diffraction grating [5,6,7,8]. Infrared (IR) frequencies from 300 GHz to 400 THz (wavelengths from 1 mm to 700 nm) have been applied in many industrial, scientific, military, and commercial fields that include imaging [9], sensing [10], and biomedical applications [11]. Therefore, the need for beam steering technology has also increased in these infrared optics-based fields, and recently, ultra-thin and fast optical devices have been in demand. For this purpose, the microelectromechanical system (MEMS), in which micromirrors are swiveled by electrostatic forces, is typically used for steering infrared-frequency beams [12,13]. However, an immediate response was not obtained due to the slow operational speed of MEMS [14,15]. As an alternative to structural tuning, metasurfaces with tunable properties are widely studied [16,17].

Two-dimensional (2D) materials, graphene, or transition metal dichalcogenides (TMDCs), such as molybdenum disulfide (MoS_2_) and tungsten selenide (WSe_2_), have atomic thicknesses and various mechanical, chemical, and optical properties [18,19]. In the case of graphene, a gate-dependent optical transition [20] could be generated under an electric field by simply controlling a current [20] to control the light transmitted from a source. Because of this gate-tunable property with a thin structure, graphene is widely used in electrically tunable optical devices [16,21,22].

Surface plasmon polaritons (SPPs) are quasi-particles generated by the interaction of light with free electrons on a metal surface. SPPs can propagate along the subwavelength scale metal surface [23]. They are actively used in nanocavity [24] and metasurface [25] research owing to their strong interactions with light. In addition, the strong interaction characteristics of SPPs allow light to interact with 2D materials that show limited optical interactions with light inherently due to their atomic thickness.

Metamaterials are artificial materials that can optically control electromagnetic waves. They have been actively researched because they have permittivity and permeability that do not exist in natural materials [26,27,28]. The metasurface, as a 2-dimensional application of metamaterials, has the unique artificial optical properties of deep subwavelength thickness. On the other hand, it was reported that metasurfaces with all-metal or all-dielectric structures could be fabricated [29,30,31,32]. Metasurfaces have been proposed for various applications, including antenna-sensor antenna [33], electromagnetic filtering [34], environmental sensing [35], and gain enhancement [36]. Antenna-sensor antenna have been demonstrated as tunable terahertz filters/antenna-sensors using graphene-based metamaterials [33]. Electromagnetic filtering has been observed in the all-metal wideband frequency-selective surface bandpass filter for different polarizations [34]. Thanks to smart metasurfaces, environmental sensing has been also studied through the advancement and artificial intelligence approaches in antennas [35]. In many antennas using different types of metasurfaces, gain enhancement has been reported [36]. Various forms of research have been studied to combine graphene with the surface plasmon of metals by placing metal structures on top of graphene [37,38,39].

Accordingly, we propose a tunable metasurface structure that could exhibit a fast-operating speed through voltage bias control using graphene in the metal gap structure. The proposed structure shows the operations of an electrically tunable metasurface mirror for dynamic beam steering and controlled focusing with a designed spatial distribution of the Fermi energy on the graphene, which is demonstrated by finite element method (FEM) simulations. It is possible to react at high speed [40,41] and immediately change the beam steering and focus by controlling the graphene bias. We anticipate that the proposed structure can overcome the limited operational speed of MEMS.

## 2. Structure Design

**Figure 1:**
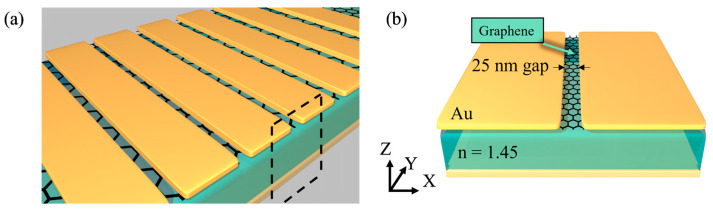
Schematic of the (**a**) electrically tunable metamaterial mirror and (**b**) unit cell of the metamaterial structure. Black dashed square in (**a**) represent the unit cell of mirror, which is shown in detail in (**b**). Au strips form a 1D array along the *x*-axis with a 25 nm gap. The single-layer graphene is placed between the arrayed Au strips and a dielectric spacer. The spacer has a thickness of 1000 nm and a refractive index of 1.45. The bottom, covered by Au, acts as a mirror as well as a common electrode.

The metasurface has been demonstrated to enable beam steering by using the concepts of phase-shifting surfaces [42] and leaky waves [43]. The structure we propose is a tunable metasurface mirror that controls beam steering through the tuning of graphene’s Fermi level based on a phase-shifting surface. We propose a tunable metasurface consisting of 1D arrayed metal (gold) strips and graphene in the thin gap between the strips, as shown in Figure 1. The gold strips with a thickness of 30 nm and a width of 1200 nm are periodically placed along the *x*-axis with 25 nm gaps between the strips. Underneath the gold strips, graphene is placed on a dielectric spacer with a thickness of 1000 nm, and the backside of the spacer is covered by gold. The backside gold substrate functions as a light reflector as well as a common electrode. Each top gold strip is biased so that the electric field can be applied to the graphene below the strip. For mechanical support, an additional substrate structure is required under the gold substrate. However, if the gold substrate is thicker than hundreds of nanometers, the electric fields cannot penetrate the gold substrate, and the additional substrate does not affect the optical property. Therefore, the additional substrate is neglected to estimate the optical performances of the proposed structure in the simulation.

The top Au strips act as an optical resonant scatterer, in which a metal–insulator–metal (MIM) plasmonic resonance gap mode appears at the gap between two Au strips [44]. The electric field enhanced in the gap is overlapped with the graphene layer. The gap mode provides a strong interaction channel in which the incident light can be more interactive with the graphene layer.

The dielectric function of the graphene layer can be expressed by ϵ=1+iσ/ωd, where σ, ω, and d are the optical conductivity, angular frequency of light, and thickness of graphene, respectively. The optical conductivity (σ) for the graphene layer is calculated according to the local random phase approximation (RPA) method, with an assumption carrier mobility μ that is 10,000 ${\mathrm{cm}}^{2}/V\cdot s$ [37,45]. According to the local RPA method, σ can be expressed by the following equation:

(1) $$\sigma =\frac{2e^{2}\omega_{T}}{\pi \hbar }\frac{i}{\omega +i\tau^{-1}}\log\left[2\cosh\left(\frac{\omega_{F}}{2\omega_{T}}\right)\right]+\frac{e^{2}}{4\hbar }\left[H\left(\frac{\omega }{2}\right)+i\frac{2\omega }{\pi }\int_{0}^{\infty }\frac{H\left(\frac{\omega^{\prime }}{2}\right)-H\left(\frac{\omega }{2}\right)}{\omega^{2}-\omega^{\prime 2}}d\omega \prime \right]$$

where $H\left(\omega \right)=\frac{\sinh\left(\omega /\omega_{T}\right)}{\cosh\left(\frac{\omega_{F}}{\omega_{T}}\right)+\cos h\left(\frac{\omega }{\omega_{T}}\right)}$, $\omega_{F}=E_{F}/\hbar$, $\omega_{T}=k_{B}T$, e is the charge of the electron, τ is the Drude relaxation rate, $E_{F}$ is the Fermi energy level, T is the temperature, $k_{B}$ is the Boltzmann constant, and ℏ is the Dirac constant. Because the Drude relaxation rate $\tau =ev_{F}^{2}/\mu E_{F}$, is actually $E_{F}$-dependent, σ can be tuned by the Fermi energy $E_{F}$ at stationary ω [46,47]. In other words, the dielectric function of a graphene layer can be controlled by the Fermi energy. Additionally, the Fermi level in Graphene $E_{F}$ can be expressed in $E_{F}=\hbar \nu_{F}{\left(\pi n_{2D}\right)}^{\frac{1}{2}}$ with $n_{2D}$ for the carrier density of graphene, which is linearly proportional to electric bias on graphene [48]. Hence, in practical devices, the electric bias can adjust the Fermi energy of graphene and, thus, its dielectric function [49,50]. In this structure, bias voltage in the graphene layer is applied by a gold strip with a back gold substrate as a common ground [16].

The simulations were performed using the 2D finite element method (FEM) tool(COMSOL Multiphysics). We constructed a metasurface mirror consisting of infinite unit-cell arrays (Figure 1b) with periodic boundary conditions. On the unit cell of Figure 1b, the 4λ thickness air domain was placed for mode profile observation. For beam steering and focusing, in Section 3.2, we used 20-unit cell arrays for steering and 51-unit cell arrays for focusing. Additionally, we included a 3000 nm thickness PML layer around the finite cell arrays. A linearly x-polarized plane wave was assumed to be the incident light normal to the surface of the proposed structure. When simulating a photonics device with a mono or a few layers of graphene—the cause of the atomic level thickness of the graphene—the macroscopic optical properties cannot be applied to the simulation directly. We modeled graphene using surface current density, and the graphene was treated as a 2D layer without any thickness. When the thickness of graphene, 0.34 nm, was much smaller than the wavelength, the model produced exactly the same optical behavior. The current density can be controlled by bias voltage [51,52].

## 3. Results

First, we investigated the reflectance and phase shift of the reflected light in the proposed infinitely periodic metasurface as a function of the wavelength and different Fermi levels at infrared wavelengths. Next, we showed the operation of beam steering in the metasurface by applying different bias voltage sets on each strip as suggested by the spatial phase change. In addition, the metasurface could focus the reflected light at the on-demanded focal point.

### 3.1. Reflectance and Phase Shifting in a Unit Cell of the Proposed Metasurface

**Figure 2:**
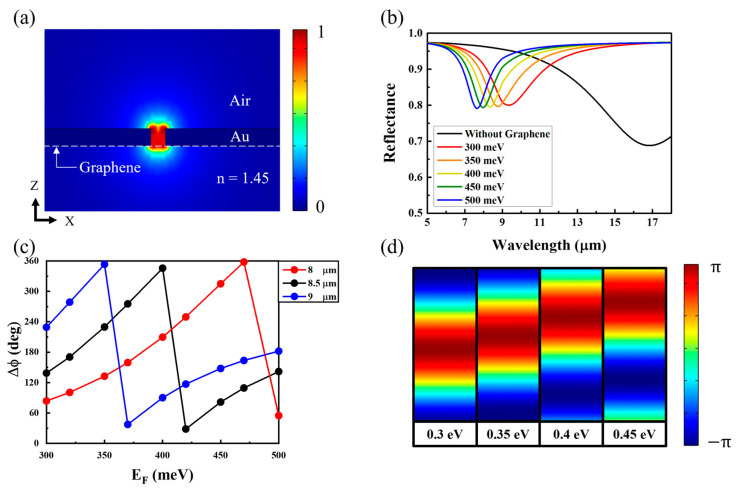
(**a**) Normalized electric field intensity profile of the MIM plasmonic mode in the XZ plane. The electric field is strongly confined in the gap between two gold strips. (**b**) Reflectance spectra for different Fermi levels on graphene and the structure without graphene (black). (**c**) Phase changes of the reflected light as functions of the Fermi level (*E_F_*) for different wavelengths of the incident light, 8 μm (red), 8.5 μm (black), and 9 μm (blue). (**d**) Normalized electric field intensity profiles of the standing wave of the incident and reflected lights (with a wavelength of 8.5 μm) for different Fermi levels from 0.3 eV to 0.45 eV.

When an Ex-linearly polarized light was normally incident on the metasurface, a strong resonance could be observed at the gap between the gold strips (Figure 2a), called the MIM plasmonic gap mode [23]. The gap mode shows a broad dip near 16.8 µm in the reflectance spectrum (black curve) shown in Figure 2b, because the resonance increased the metallic absorption of the incident light. Although the atomic thickness of graphene limits the interaction with light because of the MIM gap mode, the graphene placed at the gap can change the resonant wavelength, linewidth, and reflectance of the MIM gap mode. Moreover, the proposed metasurface structure consisting of the gold strip, graphene film, and gold substrate could be considered an effective ultrathin film with deep subwavelength thickness. Meanwhile, the deep-subwavelength ultrathin highly lossy film can cause a loss-induced large phase shift [53]. In the proposed structure, graphene provides strong absorption at the target wavelength by combining the metal gap structure; thus, a strong absorption-induced phase shift can be exhibited. When the Fermi level of graphene was 300 meV, the resonant dip (red curve) could be observed at 9.3 μm, and the lowest reflectance was 0.80. The reflectance peaks blue-shifted as the Fermi Energy (*E_F_*) increased.

To confirm the properties of the reflected light, we investigated the phase change of the reflected light as a function of the Fermi energy for three wavelengths, 8 μm (red), 8.5 μm (black), and 9 μm (blue), corresponding to the dips of the MIM plasmonic mode with the biased graphene, as shown in Figure 2c. As *E_F_* changed from 300 meV to 500 meV, the phase change of the incident light with a wavelength of 8.5 μm was covered from 0 to 360°. For the incident wavelengths of 8 μm and 9 μm, phase changes of 320 degrees were estimated as the Fermi energy was controlled. Since the phase change of the 8.5 μm light could be controlled fully over 360 degrees by manipulating the Fermi energy, the required reflected phase change φ could be determined according to the specific Fermi energy on the graphene.

Figure 2d shows that the electric field antinode of the standing wave between the incident and reflected lights moved away from the surface of the proposed metasurface as the Fermi energy increased from 300 meV to 450 meV because the phase change of the reflected light increased with the Fermi energy in Figure 2c. According to the mode profiles, the phase of the reflected light changed continuously with the Fermi energy of graphene. The Fermi energy could be controlled by applying a proper external bias onto the graphene.

### 3.2. Constructing a Metasurface Mirror with a Finite Unit Cell Array

By applying a certain Fermi energy on the graphene in the unit cell of the proposed metasurface, the phase change of the reflected light could be controlled over 360 degrees as required. Here, we limited the target wavelength to 8.5 μm. The target wavelength, which can cover the phase change of 360 degrees, can be adjusted by tunning the proposed metasurface.

**Figure 3:**
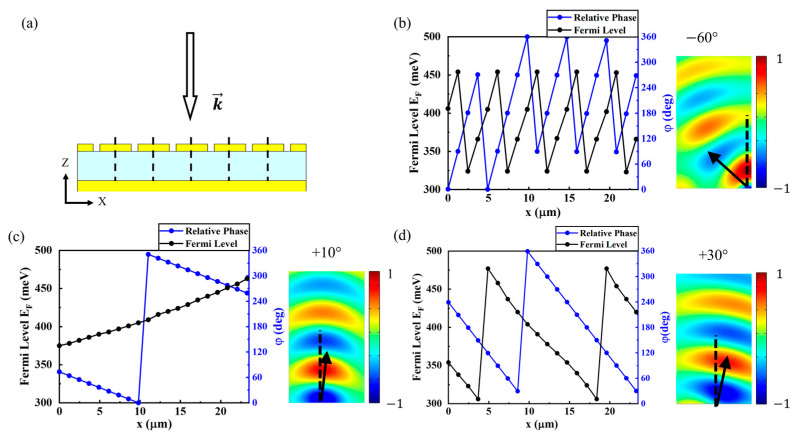
(**a**) Schematic of the tunable graphene-based metasurface mirror. Light is incident from the -z-direction, and each unit cell has a different bias voltage, i.e., Fermi energy, in the graphene layer. (**b**–**d**) Graphs of applied Fermi Energy of graphene and the corresponding reflection phase shift along the *x*-axis. Reflected electric field profiles, which are the phases of the steered beam after the reflection from the designed metasurface mirror. By applying a different Fermi energy on the graphene in each unit cell as shown in left side of (**b**–**d**), the reflected beam was steered by (**b**) −60°, (**c**) +10°, (**d**) +30° for the wavelength of 8.5 μm. The dotted line is the normal axis of the unit cell array.

Accordingly, we constructed the electric tunable metasurface mirror consisting of finite numbers of unit cell arrays, such as Figure 3a, which is functionally similar to the optical phased array mirror. By applying different biases on each unit cell and, thereby, different Fermi energies, the reflection angle became electrically tunable. Figure 3b–d shows the relation of the shifted phase and Fermi level by position, as well as propagating the electric field profiles of the steered beam after reflection for the steered angle of −60, 30, and 10 degrees, respectively.

For controlling the reflected beam direction, the reflected phase per each unit cell should be calculated. As a beam steering modulation, all reflected beams from each cell should propagate the same reflection angle, *θ*, from the incident light angle. So, in a continuous structure, the steered light phase φ(x) can be expressed in the following equation [54].

(2) φ(x)=(2π/λ)x sin θ

In this case, however, unit cells barely act as a direct beam reflector but as a reflection phase shifter. Since reflected light in the unit cell was assumed to propagate in a normal direction, we constructed beam steering through an array of shifted phases.

Meanwhile, Figure 2c shows that the reflected light phase shift can correspond to certain graphene biases. The result in Figure 2c, $\varphi_{shift}$ at 405 meV is $\varphi_{shift}=360{}^{\circ}$, which becomes the starting point of the whole beam steering structure. Next to the point, the following cells have the specific Fermi levels corresponding to the next equation. The steering angle $\theta_{steer}$ and shifted phase $\varphi_{shift}$, $\varphi_{shift}$ can be expressed in the following equation.

(3) $$\varphi_{shift}=\left(2\pi /\lambda \right)x\;\tan\left(\theta_{steer}\right)$$

By constructing the successive, tilted mode profile on the arrayed cell, beam steering could be implemented.

**Figure 4:**
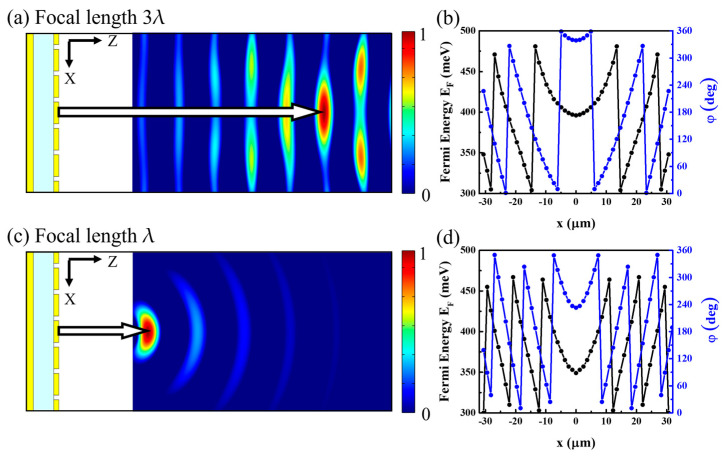
For an incident light with a wavelength of 8.5 μm, (**a**,**c**) show the reflected light with electric field profiles focused on *f* = 3λ and λ, respectively. (**b**,**d**) show the graphs of the applied Fermi energy and the corresponding reflected phase shift along the *x*-axis corresponding to (**a**,**c**). By controlling the Fermi energy on the graphene at each cell of the metasurface, the focusing position of the reflected light can be easily changed.

Therefore, the reflected beam can be propagated as intended (see Figure 3b–d) by applying a bias corresponding to φ(x). In order to maintain the plane-like shape of the wavefront of the reflected light, the maximum steering range is expected to be ±60°, which is a range large enough to be applied as a tunable concave mirror, similar to Figure 4.

From the structural limitation of the optical phased array, the missing phase range, which causes interference between nearby unit cells, deteriorates the beam steering ability by constructing side lobes [55]. However, each unit cell in the proposed structure is nearly λ/7 the size of the target wavelength, much smaller than λ/2, which is small enough to operate as a continuous phase modulator based on Fraunhofer diffraction [56,57].

As a beam-focusing modulation, all the reflected beams from each unit cell must propagate to the focal point (focal length: f). Therefore, the reflected light phase φ(x) can be expressed as follows [54]:

(4) $$\varphi \left(x\right)=\left(2\pi /\lambda \right)\;\sqrt{x^{2}+f^{2}}-f$$

To modulate beam focusing as expected, the shifted phase should also be placed as parabolic. Hence, $\varphi_{shift}$ can be expressed in the following equation.

(5) $$\varphi_{shift}\left(x\right)=\frac{\left(\frac{2\pi }{\lambda }\right)\left(\sqrt{f^{2}-x^{2}}-f\right)\cdot f}{\sqrt{f^{2}+x^{2}}}$$

Furthermore, by applying a different Fermi energy, corresponding to Equation (5), beam focusing can be achieved, as shown in Figure 4, for different focal lengths of 3l and l.

## 4. Conclusions

In this paper, we propose a graphene-based electrically tunable metasurface mirror for dynamic beam steering and focusing. In this approach, the Fermi energy of graphene in each unit cell in the metasurface was designed to realize beam steering and focusing.

For the incident light, two gold strips, placed on a low-index dielectric with nanogaps of 25 nm, confined the electric field due to the MIM plasmonic gap resonance, resulting in an absorption dip of the reflectance spectrum at infrared wavelengths. The graphene between the gold strips experienced a strong field such that the change in the Fermi energy induced a resonant wavelength shift. For an incident light with a wavelength of 8.5 μm, the phase of the reflected light could be tuned from 0 to 360° by controlling the Fermi energy from 300 meV to 500 meV. Further, the reflectance was larger than 80% for the operating wavelength.

Based on the phase change property of the reflected light on the proposed metasurface unit cell, the reflected light of a normally incident light can be steered as required when the spatial distribution of the Fermi energy of the whole metasurface is designed to induce a phase shift of the angled reflected light. Beam steering of −60°, +10°, and +30° was demonstrated using this proposed approach. In addition, the reflected light could be focused at one point, and the focal length could be tuned from one wavelength, λ, to three wavelengths, 3λ.

The fabrication of metal-strip/dielectric/metal structures with graphene has been reported by various researchers. According to Guo, Xuguang et al. (2021) [39], graphene was attached under a metal grating using polymethyl methacrylate (PMMA), and two benzocyclobutene (BCB) was used as a spacer. Although there is a difference in scale, the process of our structure is expected to be sufficiently feasible. In addition, Gahoi, Amit et al. (2016) [38] showed that it is possible to place graphene on a silica spacer and deposit metal on top of it by using PMMA as a transfer medium with the same structure as a back gate on a silicon substrate. Additionally, Naresh K. Emani et al. (2012) [37] showed that it is possible to realize metamaterials by placing graphene on a dielectric spacer and placing a metal scatterer of hundreds of micrometers on top of it. Therefore, the proposed structure is also fully realizable by growing a dielectric spacer on a metal mirror, transferring graphene through PMMA, and then depositing metal strips.

The 25 nm or smaller gap can be fabricated by various methods, such as focused ion beam milling (FIB) [58,59], e-beam lithography (EBL) [60,61], and atomic layer deposition (ALD) [62,63].

Recently, Yazdi, G. Reza et al. (2013) and Wang, M et al. (2021) reported the fabrication of 50 × 50 μm^2^ over large-scale monolayer graphene [64,65]. In the proposed structure, the entire size of the metasurface for beam steering and focusing were 24 μm and 64 μm. Hence, we expect that our proposed structure can be fabricated.

Although graphene is highly utilized due to its electrical tunability, thermal expansion damage is likely to occur during the deposition process on the substrate. Therefore, it is necessary to manufacture devices that consider the difference in thermal expansion.

Beam steering of the metasurface can be measured using the free-space Michelson interferometer [66].

Table 1 is a comparison table between our structure and other tunable metasurfaces. The proposed structure numerically confirmed that it could be applicable to beam steering ± 60 degrees with the electrical control of graphene’s Fermi level.

In conclusion, we performed relatively straightforward numerical simulations and demonstrated that dynamic beam steering and focusing can be implemented by applying different spatial distributions of the Fermi energy of graphene in the proposed metasurface. The proposed electrically tunable metasurface mirror can be applied to mid-IR optics and photonics for astronomy, IR imaging, and chemical sensing owing to its fast response and high reflectance [67,68].

## Figures and Tables

**Table 1:** Comparison table of the tunable metasurfaces.

Structure	Mechanism	Wavelength	Material	Function
Our Structure	Electrical Tuning	8.5 μm	Metal/Graphene	Beam Steering
Atwater et al. (2016) [16]	Electrical Tuning	1550 nm	Metal/Dielectric	Beam Steering
Zhou et al. (2019) [17]	Temperature Tuning	25–60 μm	Metal/Dielectric	Reflectance Tuning
Lalbakhsh et al. (2021) [29]	Mechanical Tuning	60 mm	All Metal	Gain Enhancement

## Data Availability

Data is contained within the article.
